# Modelling the contribution of walking between home and school to daily physical activity in primary age children

**DOI:** 10.1186/s12889-015-1765-7

**Published:** 2015-05-01

**Authors:** Rebecca M Stanley, Carol Maher, James Dollman

**Affiliations:** University of Wollongong, Early Start Research Institute, School of Education, Northfields Ave, Wollongong, 2522 New South Wales Australia; University of South Australia, Alliance for Research in Exercise, Nutrition and Activity, School of Health Sciences, GPO Box 2471, Adelaide, 5001 South Australia Australia

**Keywords:** Active transportation, Children, Physical activity, Accelerometry

## Abstract

**Background:**

The purpose of this study was to identify the independent association of frequency of walking trips between home and school with daily physical activity in a sample of school-aged children.

**Methods:**

Participants were 109 children (mean age = 12.05 years [±0.71]) attending nine primary schools in Adelaide, South Australia. Physical activity was derived from accelerometers with total counts as the outcome variable. Transport patterns were self-reported for each of the previous five school days. Walking trips were summed for each day and across the school week. The relationship between the number of active transport journeys and individual school day and school week physical activity was modelled separately in boys and girls using multiple linear regression.

**Results:**

Frequency of walking was positively associated with school day and school week accelerometer counts in boys, accounting for 6% and 12% of the explained variance in total counts, respectively. There were no significant associations among girls.

**Conclusion:**

Despite sex-specific differences in associations between active transport to school and total physical activity, active transport is likely to have important ancillary benefits for development of independence and physical activity habits, and should continue to be promoted.

## Background

There is now convincing evidence for the health benefits of regular physical activity among young people, including improved cardiovascular fitness, body composition, and resting blood pressure [1,2]. Promoting physical activity among young people is a serious public health challenge, especially given the cultural shifts and changes in home and neighbourhood environments that encourage sedentary behaviours and discourage physical activity [3]. It has been proposed that children are more likely to maintain an adequate physical activity level if additional activity is integrated into everyday life, such as active transport [4].

Active transport (AT), particularly walking and cycling between home and school, has been identified as a potential means of embedding a regular ‘dose’ of physical activity on most days of the year [5,6]. Evidence shows that children who walk to and from school are more active in the mornings and afternoons compared to those who ride in a car or bus [4,7]. A recent systematic review found consistently strong evidence of a positive association between active travel and overall physical activity [8]. However, what is still unclear from the current evidence is the magnitude of the contribution of AT trips to daily physical activity levels and the impact of higher frequency of active commuting [9]. Although many studies have explored the association between AT and physical activity levels [8], the majority have categorised children by travel mode (e.g. active versus inactive travel) and then compared overall physical activity levels between categories [10]. Such studies have found that children who are ‘active commuters’ between home and school can accumulate an additional 20 minutes of moderate-to-vigorous physical activity (MVPA) across the whole day compared to children who are driven [11,12]. However, the limitation with categorising participants into commuting categories is that dose-response relationships between the number of active trips and physical activity are obscured [4]. To date, it remains unclear how the frequency of active journeys in, say, a day or a week relates cumulatively to overall physical activity. Further, some studies have shown positive relationships between AT and total physical activity in boys but not girls [13,14], while other studies have not reported sex-specific relationships [15].

Accordingly, the purpose of the current study was to identify the independent associations of frequency of walking trips between home and school with daily physical activity (range of trips 0 - 2) and total physical activity across four school days (range of trips 0 - 8) in a sample of upper primary school aged children, stratified by sex.

## Methods

### Participants

Participants were recruited from government and independent primary schools in Adelaide, South Australia. Recruitment material was sent to twenty randomly selected urban schools, and nine schools agreed to participate in the study. Participant information packages were sent to Year 6 and 7 students in participating schools. Written informed consent and verbal assent were obtained from parents and children, respectively. The study was approved by the University of South Australia’s Human Research Ethics Committee.

### Physical activity

Physical activity was measured using Actigraph 7164 accelerometers (formerly known as the Computer Science and Applications, Inc [CSA] 7164). The Actigraph accelerometer is a common objective measure of physical activity and the CSA 7164 has been accepted as a valid and reliable tool to measure physical activity in adolescents [16]. The accelerometer was worn on an adjustable elasticised belt around the waist, over the right hip, anterior to the iliac crest [17]. A one-minute epoch interval was used to be consistent with other studies [14,17,18]. Valid days comprised a minimum of 10 hours of ‘wear time’ [19]. Total counts were used as the outcome variable as there are no universally accepted cut-points for physical activity intensities in the literature [20] and continuous measure of physical activity can be more sensitive than categorical variables [21].

Participants wore the accelerometer for 8 consecutive days (Wednesday to Wednesday), removing only for sleeping, showering or swimming. This allowed inclusion of four complete school days (Monday, Tuesday, Thursday and Friday) in the analyses. Data were prepared for the following school day segments, based on each school’s bell times: whole day, before school, recess, lunch time, after school.

### Active transport

Participants self-reported transport patterns for each of the previous five school days in the categories “walked”, “rode bike”, “car, bus or train”; and “did not go to school”. Days on which children were absent from school or cycled were removed on the basis that accelerometry is relatively insensitive to cycling. The walking trips on days corresponding with full accelerometer data (i.e. Monday, Tuesday, Thursday and Friday) were summed for each day (possible scores: 0, 1, and 2) and across the school week (possible scores: 0-8).

Discriminatory validity of the AT questionnaire was determined by regressing self-reported walking trips to school (yes/no) against accelerometer counts accrued in the before school period, and then again with accelerometer counts accrued during the lunch period. Results showed that the walking journey to school was consistently associated with before school accelerometer counts in both boys and girls on all days, except boys on Thursday mornings (R^2^ ranged from 8 - 43%, p < 0.05), and was consistently not associated with lunchtime accelerometer counts, providing evidence of the tool’s discriminatory validity (data not shown).

### Height and weight

Height was measured without shoes to the nearest 0.1 cm using a portable stadiometer (Surgical and Medical Products, Australia) and weight was measured to the nearest 0.1 kg using a digital laboratory scale (HoMedics, Inc, Model # SC-560, Australia). Body mass index (BMI) was calculated as weight (kg) divided by height (m) squared, and BMI z-scores were calculated according to Cole et al. [22,23].

### Socio-economic status

For each individual participant, area level socio-economic status was determined from residential postcode, based upon the Australian Bureau of Statistics (ABS) Socio-Economic Indexes For Area (SEIFA) Index of Relative Socio-Economic Disadvantage [24]. The national average is set at 1000 (±100), with higher values representing higher socioeconomic status. A SEIFA score was assigned to each participant based on his or her postcode of residence.

### Statistical analysis

Analyses were limited to children who provided compliant accelerometer data on four school days and at least one weekend day, and the completed AT questionnaire. Descriptive analyses summarised the demographic data (age, BMI and SEIFA score) and mean activity counts for boys and girls.

The relationships between AT journeys and daily physical activity were explored within individual school days and for the school week. For the school day models, data from each of the four days of measurement were pooled to form a dataset of 436 individual days (i.e. school day was the unit of analysis). Multiple regression models were established with daily accelerometer counts as the dependent variable and the number of walking journeys (0, 1 or 2) as the independent variable, separately in boys and girls. Adjusted models were also constructed, controlling for age, zBMI, SEIFA and weekend accelerometer counts. Weekend accelerometer counts was included as a covariate as it could be argued that children who actively commute to and from school are generally more active than those who do not. As each child contributed four days of data to the pool, clustering by child was accounted for in the regression models with robust standard errors.

For school week models, the child was the unit of analysis. Multiple regression models were established with average school day accelerometer counts as the dependent variable and the total number of walking journeys (0-8) in the four monitored days as the independent variable, separately in boys and girls. Adjusted models were also constructed, controlling for age, zBMI, SEIFA score and weekend accelerometer counts. Clustering by school was accounted for in the regression models with robust standard errors. Statistical significance was inferred at p ≤ 0.05 in all models. All analyses were conducted using STATA v12 (College Station, Texas).

## Results

### Participant characteristics

A total of 213 children (93 females, 120 males) took part in this study with a response rate of 52%. Of the 213 children, 109 children (58 females, 51 males) provided sufficient accelerometer data and a completed AT questionnaire. There were no significant differences between boys and girls in age, BMI z-scores, socio-economic status (SES), average weekend day counts or the frequency of walking trips. However, boys were significantly more active than girls on weekdays (p = 0.04). Also, there were no significant differences between those who did and those who did not provide valid accelerometry data, for age, gender, zBMI and SEIFA. Table 1 summarises the demographic characteristics of the sample. The mean SEIFA score for participating children were 1034.5 (SD 69.20) for boys and 1031.3 (SD 67.13) for girls, suggesting that the sample comprised children who typically came from above average socio-economic status backgrounds.

**Table 1:** **Demographic characteristics of the sample of South Australian children (means [SD])**

	**Males (n = 51)**	**Females (n = 58)**
Age (years)	12.00 (0.69)	12.10 (0.72)
zBMI	0.79 (1.02)	0.47 (1.19)
SEIFA^a^	1034.5 (69.20)	1031.3 (67.13)
Average weekday counts	565472 (154987)	481222 (248359)
Average weekend day counts	328572 (132893)	315867 (148176)
Average number of walking trips per school week	2.29 (3.05)	2.88 (3.24)

^a^SEIFA = Socio-Economic Index for Areas score for SES levels. The nation-wide average SEIFA score is 1000 (SD = 100).

### School day models

Table 2 summarises the results from the school day regression models. Among boys, in the unadjusted school day model, daily walk number was significantly associated with whole day accelerometer counts, explaining 6% of the variance in daily activity. In the adjusted school day model, walk number, SEIFA and zBMI together explained 19% of the variance in school day accelerometer counts in boys. Among girls, there were no significant associations between the frequency of walking and daily accelerometer counts.

**Table 2:** **Unadjusted and adjusted regression models for daily physical activity**

**Variable**	**Males (n = 192 days)**				**Females (n = 232 days)**			
	**Coefficient B (95% CI)**	**Robust SE**	**Partial eta** ^**2**^	**Model R** ^**2**^	**Coefficient B (95% CI)**	**Robust SE**	**Partial eta** ^**2**^	**Model R** ^**2**^
**Unadjusted**								
Walk number	61670 (14958 – 108383)*	23257	na	0.064	32481 (-5128 – 70091)	18782	na	0.004
**Adjusted**								
Walk number	62708 (15935 – 109481)**	23250	0.068	0.19	19305 (-14304 – 52914)	16784	0.001	0.01
SEIFA	-808 (-1331 – -284)**	260	0.074		-274 (-695 – 146)	210	0.001	
zBMI	45759 (6470 – 85046)*	17798	0.051		-3552 (-52072 – 44967)	24230	0.000	
Age	20588 (-28074 – 69251)	24190	0.005		63304 (-446443 – 173052)	54806	0.008	
Weekend counts	0.22000 (0.0141 – 0.4259)*	0.1024	0.030		0.16659 (-0.00727 – 0.34045)	0.0868	0.004	

*p < 0.05, **p < 0.01, na = not applicable.

### School week models

Table 3 summarises the results from the school week regression models. For boys in the unadjusted school week model, weekly walk number explained 12% of the variance in school week accelerometer counts. In the adjusted model, weekly walk number, weekend counts, SEIFA and zBMI together explained 38% of the variance in school week accelerometer counts. Among girls, there were no significant predictors of school week accelerometer counts.

**Table 3:** **Unadjusted and adjusted regression models for average weekly physical activity**

**Variable**	**Males (n = 48)**				**Females (n = 58)**			
	**Coefficient B (95% CI)**	**Robust SE**	**Partial eta** ^**2**^	**Model R** ^**2**^	**Coefficient B (95% CI)**	**Robust SE**	**Partial eta** ^**2**^	**Model R** ^**2**^
**Unadjusted**								
Walk number	17914 (3173 – 32653)*	6392	na	0.12	9347 (-1786 – 20481)	4828	na	0.01
**Adjusted**								
Walk number	71239 (20340 – 122138)*	22072	0.136	0.38	5298 (-4092 – 14688)	4072	0.005	0.05
SEIFA	-2954 (-4849 – -1059)*	822	0.130		-320 (-787 – 158)	203	0.008	
zBMI	168410 (71171 – 265648)**	12840	0.107		-5798 (-75515 – 63918)	30332	0.001	
Age	18055 (-17600 – 53710)	15462	0.008		59797 (-59959 – 179554)	51933	0.025	
Weekend counts	0.631 (0.297 – 0.966)**	0.145	0.117		0.17803 (-0.9957 – 0.45564)	0.12038	0.008	

*p < 0.05, **p < 0.01, na = not applicable.

## Discussion

To our knowledge, this study is the first to explore whether the frequency of walking trips to school is associated with objectively measured total physical activity. This study found that, among boys, AT explained 6% and 12% of variation in total physical activity across the school day and school week respectively, while there were no associations among girls.

Two other studies have reported an association of AT with total physical activity in boys but not girls: Carver et al. [13] found that AT to and from school was associated with weekday MVPA in boys but not girls, while Cooper et al. [18] found that boys who walked to school were more active overall than those who were driven to school, a relationship again not seen in girls. The reasons for these sex-specific differences are currently unclear. Given that girls undertake less total daily physical activity than boys, one might expect AT trips that are the same distance for all children to contribute relatively more to total daily physical activity in girls than in boys. Alternatively, attributes of the AT journey itself may contribute to sex-specific patterns, such as the speed of walking and the load of schoolbags and/or sports equipment relative to muscle mass [25].

Previous researchers in the field have cautioned that positive associations between AT and total physical activity may simply reflect active children’s preference to actively commute between home and school, or that it may prompt children to be more active at other times of the day, rather than the direct contribution that AT makes to total physical activity [15,18]. This hypothesis was accounted for in the current study by adjusting analyses for weekend activity [18]. We found that, even after accounting for weekend physical activity, AT journeys among boys were associated with school day physical activity, within single days and across a school week. This finding suggests that the AT journeys directly contribute to boys’ daily physical activity. In support of this, Abbott et al. [15] found no difference in weekend step counts between children who walk to school and those who were transported passively.

Some limitations should be acknowledged when interpreting the results of this study. Only self-reported transport mode was collected with no details of potentially informative characteristics such as walking speed, load burden and distance travelled. Previous research has shown that the contribution of AT to physical activity is greatest in children undertaking longer journeys [26-28]. However, there is evidence to suggest that active journeys are more likely to be within an 800 m radius [29,30], while passive journeys are more likely beyond this threshold. Despite this, it is still important to acknowledge the lack of a measure of distance is a limitation of the study. The sample size was modest, thus there is a risk of type 2 errors (not detecting a relationship that truly exists). However, the results did not suggest trends in the data approaching statistical significance, thus it seems unlikely that the lack of association in girls was a product of the sample size. A strength of this study was the use of objectively measured physical activity. Additionally, analyses based on the accelerometry data were undertaken using total activity counts, rather than user-defined cut-points, which can overestimate or underestimate children’s physical activity [31]. While accelerometers are the most common objective measure of physical activity, it is acknowledged that they do not provide valid estimates of physical activity during cycling, hence the removal of cyclists from the analysis. Future studies should implement alternative methods to capture physical activity obtained from cycling. In addition, the self-report measure of active transport used in this study took a one-dimensional view of AT and did not consider emerging AT modes commonly used by primary school aged children, such as skateboarding and scootering, which can also result in health improving energy expenditure.

The findings from this study have highlighted a number of future recommendations. The application of geographic positioning systems (GPS) and geographic information systems (GIS) in future studies would shed light on AT journey characteristics, such as timing, speed, distance, geographic contour of the route and mixed modality (e.g. walking to the bus stop). Direct weighing of school bags and other cargo, along with body weight, would allow as estimate of sex-specific stresses of load.

## Conclusion

AT between home and school contribute a reasonably small proportion of daily physical activity. Among boys, but not girls, AT is associated with total physical activity. However, the public health importance of AT to school may go beyond these associations by contributing to the broader habit of AT for other reasons and to other destinations [32]. Furthermore, unescorted trips to and from school allow children to develop their independent mobility, which may contribute to children’s social, emotional and cognitive development [33]. Accordingly, continued promotion of AT between home and school is warranted. Into the future, application of innovative technologies will allow important movement and spatial characteristics of the journey to be better understood.
